# RNA isolation method for single embryo transcriptome analysis in zebrafish

**DOI:** 10.1186/1756-0500-3-73

**Published:** 2010-03-16

**Authors:** Mark de Jong, Han Rauwerda, Oskar Bruning, Jurgo Verkooijen, Herman P Spaink, Timo M Breit

**Affiliations:** 1 MicroArray Department & Integrative Bioinformatics Unit, Swammerdam Institute for Life Sciences, Faculty of Science, University of Amsterdam, Science Park 904, 1098 XH, Amsterdam, the Netherlands; 2 Department of Molecular Cell Biology, Institute of Biology, Leiden University, Gorlaeus Laboratories - Cell Observatorium, Einsteinweg 55, 2333 CE, Leiden, the Netherlands

## Abstract

**Background:**

Transcriptome analysis during embryogenesis usually requires pooling of embryos to obtain sufficient RNA. Hence, the measured levels of gene-expression represent the average mRNA levels of pooled samples and the biological variation among individuals is confounded. This can irreversibly reduce the robustness, resolution, or expressiveness of the experiment. Therefore, we developed a robust method to isolate abundant high-quality RNA from individual embryos to perform single embryo transcriptome analyses using zebrafish as a model organism. Available methods for embryonic zebrafish RNA isolation minimally utilize ten embryos. Further downscaling of these methods to one embryo is practically not feasible.

**Findings:**

We developed a single embryo RNA extraction method based on sample homogenization in liquid nitrogen, RNA extraction with phenol and column purification. Evaluation of this method showed that: the quality of the RNA was very good with an average RIN value of 8.3-8.9; the yield was always ≥ 200 ng RNA per embryo; the method was applicable to all stages of zebrafish embryogenesis; the success rate was almost 100%; and the extracted RNA performed excellent in microarray experiments in that the technical variation was much lower than the biological variation.

**Conclusions:**

Presented is a high-quality, robust RNA isolation method. Obtaining sufficient RNA from single embryos eliminates the necessity of sample pooling and its associated drawbacks. Although our RNA isolation method has been setup for transcriptome analysis in zebrafish, it can also be used for other model systems and other applications like (q)PCR and transcriptome sequencing.

## Background

Transcriptome studies of model organisms during development such as *Mus musculus *[1], *Drosophila melanogaster *[2] and *Caenorhabditis elegans *[3] are exciting research fields with many opportunities, yet often hampered by the availability or size of biological materials. In the last two decades Zebrafish (*Danio rerio) *has joined these experimental model organisms in many domains of biological and biomedical research [4-6]. This is also fueled by the convenient morpholino technique, in which antisense oligonucleotide injection -usually in eggs or embryos- effectively 'knocks down' target gene expression [7]. Additionally, the morpholino technique in combination with genome-wide transcriptome analysis has boosted developmental studies involving zebrafish embryogenesis. Hence, many research groups worldwide embraced this model system in their research.

In studies of genetic networks in zebrafish development [8], responses to *e.g*. pathogen infection [9,10], or tissue specificity [11-14], biological materials are often pooled to obtain sufficient RNA. The results of these studies are undoubtedly of great value, but each measurement can only be interpreted as the average profile of the selected pool. Although pooling can be useful in some studies, the possible downsides of pooling are beyond discussion [15-17]. Pooling should therefore be an optional step, rather than a necessity. Especially with the rapidly developing zebrafish embryos that are only identified by broad-range phenotypic markers, pools of embryos will show variability because the embryos in it will not reside in the exact same embryonic phase. To tackle these drawbacks and adopt a more systems biology approach aimed at individual systems, we developed a reliable protocol to isolate high-quality RNA from individual zebrafish embryos with a yield sufficient for microarray analysis and other transcriptome analysis techniques.

Available methods for embryonic zebrafish RNA isolation commonly need to utilize 20 embryos or more to obtain sufficient RNA (For examples see *The zebrafish book *[18] and [19]). Scaling extraction volumes proportionally down to less embryos, is feasible to ~10 embryos, but smaller numbers lead to unworkable methods. One method is described to isolate RNA from 10 embryos at 36 hpf (~500 ng/embryo) or 52 hpf (~600 ng/embryo) [13], but as these embryos were already relatively far in development, it was not clear if this would also be applicable to earlier stage embryos. In this work, several methods were investigated for isolating RNA from individual embryos with the following prerequisites: 1) Isolated RNA should display a RNA Integrity Number (RIN, quality measurement from Agilent Technologies) greater than or equal to 7.0; 2) The RNA yield per embryo should be sufficient to allow quality checks and downstream processing, i.e. more than 200 ng; 3) Impurities should be low, so that mRNA amplifications can be performed.

One of the challenges of isolating RNA from zebrafish is the presence of a rigid and insoluble chorion. Enzymatic degradation of the chorion is possible, but will most likely affect the transcriptome. Therefore, we chose for a sample preparation method involving mechanical disruption of the chorion under frozen conditions. RNA isolations itself, can be performed by several well-established methods based on phenol-chloroform extraction and precipitation or column-based nucleic acid purification with the aid of *e.g*. guanidine thiocyanate. All kinds of methods and commercial kits are available today, each with particular advantages and applications. We have tested several published methods based on phenol-chloroform extractions with precipitation, column purifications and bead-based extraction methods. None of them performed well enough with respect to purity, yield and reproducibility. We therefore changed one of these methods [14] until it fulfilled our requirements of quality, yield and reproducibility. Important observations during the optimization were: 1) Incompletely homogenized samples gave lower quality RNA; 2) Good homogenization was only obtained with frozen samples; 3) Increased volumes of applied Qiazol lowered yield substantially and 4) The use of a phase-separation agent (*e.g*. phase-lock gel heavy) is indispensable for maximum yield and limiting Qiazol carry-over. To validate and demonstrate the value of our method, several dedicated experiments were performed.

## Results

### RNA quality and technical variation

**Figure 1:**
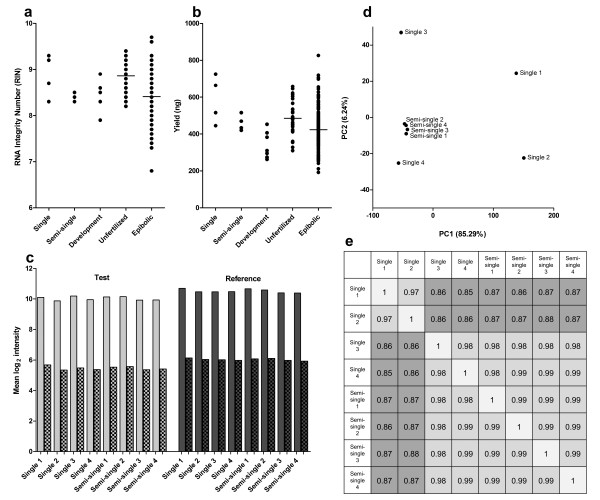
**Validation of the RNA isolation method from single zebrafish embryos**. (a) RNA RIN values and (b) yields from: Single, individual embryos (4); Semi-single, homogenized, pooled, and split embryo material (4, see text); Developmental, embryos from the 16-cell to 8-somite stage (8); Unfertilized, unfertilized eggs (30); Epibolic, embryos from dome stage to 90% epiboly (186). Stages and RNA yield could not be linked. Note that RIN values show overlap because of a single decimal place measurement accuracy. (c) Mean, unnormalized log_2 _signal intensities from microarray analysis (smooth bar, foreground signal and scatter board bar, background signal). (d) Principal component analysis (PCA) of the unnormalized log_2 _ratios (test/reference) from the Single and Semi-single samples. (e) Spearman correlations showing the similarity of the unnormalized microarray data from Single and Semi-single samples.

To investigate the technical variation of our method, RNA was isolated from eight individual embryos (*Danio rerio*, strain *AB*) at the germ ring stage. Because embryonic staging is quite difficult, the embryos will be in a marginally different stage and will because of that, show biological variation. From four embryos, RNA was individually isolated (called Single 1 to 4). To eliminate the biological variation, the other four embryos were first individually homogenized, then the homogenized material was pooled, and finally split into four samples for separate RNA isolations (Semi-single 1 to 4). Hence, the Semi-single samples should hold the same RNA content and differences could be attributed solely to technical variation of the RNA isolation method. The quality of the isolated RNA was very good, as all RIN values were ≥ 8.3 (Figure 1a) [Additional file 1]. All RNA isolations yielded ≥ 400 ng (Figure 1b). As expected, the variation in RNA yield was much lower in the Semi-single samples than the Single samples. This might indicate that RNA content varies greatly between individual embryos, although column affinity differences by, for example, higher silica content could also have caused this. To further assess the quality of the isolated RNA, the mRNA from each sample was amplified, labeled, and hybridized on a custom Agilent 8 × 15 k zebrafish microarray using standard microarray procedures. RNA isolated from a pool of 20 embryos from the germ ring stage served as a common reference RNA sample. This RNA was isolated with similar volumes as used for single embryos as described in the methods section and yielded ~350 ng/embryo with RIN 8.7. Figure 1c shows that the mean unnormalized log_2 _signal intensities of reference, as well as test samples on all microarrays were all well-above background and highly comparable. The variance in the unnormalized log_2 _ratios (test/reference) between the samples was investigated with a principle component analysis (PCA) (Figure 1d), which clearly showed that the 4 Semi-single samples clustered very close together, whereas the Single samples were widely spread. This implies that the technical variation of our RNA isolation method, even without data normalization, is much smaller than the biological variation, especially since most of the variance is observed on the PC1 axis (accounting for 85% of the variance). Finally, the Spearman correlation coefficients between the RNA isolations showed that the Semi-single samples are all highly similar (Figure 1e). The correlations between the reference samples were all ≥ 0.99 (data not shown).

### Robustness

To show the robustness of our RNA isolation methods, two experiments including larger numbers of samples were executed: 30 unfertilized eggs and 186 individual embryos, ranging from dome stage to 90% epiboly. The average RIN value of all samples was 8.9 for the unfertilized eggs and 8.4 for the epibolic samples (Figure 1a). Only one sample from all isolations was observed with a RIN value below 7.0 (Figure 1a), which is below the quality prerequisite. All samples yielded ≥ 200 ng of RNA (Figure 1b).

### Applicability in zebrafish embryogenesis

**Figure 2:**
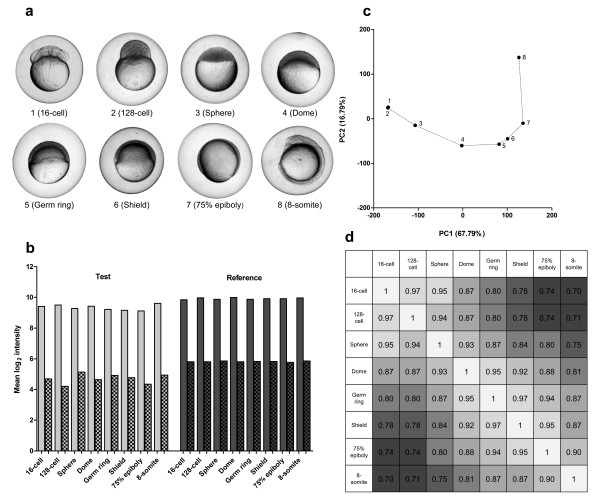
**Dissecting zebrafish development with microarray analysis**. (a) Eight selected embryos ranging from the 16-cell to 8-somite stage. (b) Mean, unnormalized log_2 _signal intensities from microarray analysis (smooth bars, foreground signal and scatter board bars, background signal). (c) Principal component analysis (PCA) on unnormalized log_2 _ratio data (test/reference) showing a 'developmental' curve starting at the 16-cell stage and ending at the 8-somite stage. (d) Spearman correlations between the samples reflect the developmental distance.

To demonstrate applicability throughout zebrafish embryogenesis, a developmental set of eight embryos was selected, ranging from 16-cell to the 8-somite stage (Figure 2a). The quality of the isolated RNA was good, as all RIN values were ≥ 7.9 (Figure 1a) [Additional file 2] and yields were all above 200 ng (Figure 1b). Pooling a small fraction of RNA from each sample constituted the common reference. All signals on the custom Agilent 8 × 15 k zebrafish microarrays were well-above background (Figure 2b) and the common reference signals were highly comparable. The PCA on the unnormalized log_2 _ratios (test/reference) showed a developmentally ordered separation of all samples (Figure 2c). The Spearman correlations showed a similar picture with samples further apart in the embryogenesis having a lower correlation. The correlations between the reference samples were all ≥ 0.99 (data not shown).

## Conclusions

In summary, here we present a robust RNA isolation method for individual zebrafish embryos. The validation of this method showed that the technical variation is much lower than the biological variation. Moreover, this method seems excellently suited to distinguish different embryonic stages by microarray analysis. Although this method has been setup with a focus on transcriptome analysis, it can also be used for other applications like (q)PCR or transcriptome sequencing. Our method could also be made applicable for embryos and small samples of dissected tissues from other model systems, as we already have done for small human skin biopsies, especially when sample material is limited or pooling is unwanted.

## Methods

### Biological materials

Zebrafish were handled in compliance with local animal welfare regulations and maintained according to standard protocols http://zfin.org[20]. Embryos of *Danio rerio *(strain *AB*) were kept at 30°C in egg water (60 μg/ml Ocean sea salts). Individual embryos were imaged with stereo microscopy (Leica MZ16 FA) and transferred to 1.5 ml tubes. Remaining egg water was removed and embryos were quickly snap frozen in liquid nitrogen and stored at -80°C. Females were anesthetized briefly in egg water containing 0.02% buffered ethyl 3-aminobenzoate methanesulfonate (Tricaine, Sigma-Aldrich). They were transferred to petri-dishes and unfertilized eggs were harvested by gentle squeezing. Afterwards, they were allowed to recover for one month.

### Zebrafish test microarrays

Microarrays have been custom designed and obtained from Agilent Technologies using the 8 × 15 k slide format. The 15 k microarray (design ID: 021987) contains ~15.000 probes and has been designed on a non-rendundant set of Ensembl and Vega transcripts with a ZFIN annotation. The design for this array and microarray data discussed in this publication have been deposited in the National Center for Biotechnology Information Gene Expression Omnibus (GEO) [21-23] and are accessible through GEO series number GSE17736 and GSE17738.

### RNA extraction protocol

Per RNA isolation, one 1.5 ml tube was filled beforehand with 75-100 mg phase-lock gel heavy (5-Prime) and pelleted for 30 s at 12,000 × g. Tubes with individual embryos were kept in liquid nitrogen until processing. Single embryos were grinded individually with a liquid nitrogen pre-chilled metal micro-pestle (Carl Roth). The pestle was lifted slightly and 200 μl Qiazol (Qiagen) was added. The pestle was placed back into the tube with Qiazol and the homogenate was allowed to thaw. Before removal, the pestle was washed with an additional 100 μl Qiazol to rinse of any material that might have stuck to the pestle. The homogenate was vortexed vigorously for 15 s, left at room temperature for at least 5 min, and then spun down quickly for 15 s. 60 μl chloroform was added to the homogenate, vortexed for 15 s and kept at room temperature for 3 min. The partly separated mixture was transferred as a whole to a pre-prepared phase-lock gel heavy containing tube and centrifuged for 15 min at 12,000 × g. The aqueous phase was transferred to a new 1.5 ml tube. The RNA was purified by column precipitation according to the RNeasy MinElute Cleanup Handbook (version 2007) - Appendix D: RNA Cleanup after Lysis and Homogenization with Qiazol Lysis Reagent (Qiagen). At the end of the procedure, the RNA was eluted in 14 μl nuclease-free water.

### RNA yield and quality

The amount of RNA per μl was measured on the NanoDrop ND-1000 (Thermo Scientific). The integrity of the RNA was investigated with the BioAnalyzer (Agilent Technolgies) using the RNA pico 6000 kit (Agilent Technologies).

### Amplification and labelling of RNA

200 ng RNA including controls, (Spikeset A for Cy3 and -B for Cy5, 1/16 final dilution) from the Two-Color RNA Spike-In Kit (Agilent Technologies), was taken as input for half volume reactions of the one-round mRNA amplification per zebrafish embryo (Amino-allyl MessageAmp II kit, Applied Biosystems). 5 μg of amplified RNA was dried in a speedvac and dissolved in 5 μl 50 mM carbonate buffer (pH 8.5) by thoroughly up and down pipetting and placed at 42°C for 5 min. They were vortexed and spun down briefly. Vials of mono-reactive CyDyes (GE Healthcare) were dissolved in 200 μl DMSO. To each reaction 10 μl of dissolved CyDye was added (Test - Cy3, Reference - Cy5). They were mixed thoroughly by pipetting up and down, vortexed and spun down briefly. Subsequently, samples were incubated for 60 min at room temperature in the dark. After 30 min the samples were vortexed and spun down briefly and left the residual 30 min in the dark. The reactions were quenched by addition of 5 μl 4 M hydroxylamine for 15 min in the dark at room temperature. 80 μl of nuclease-free water was added to each sample to bring the volume to 100 μl. The RNA was purified with column precipitation according to the RNeasy MinElute Cleanup Handbook (version 2007) - Protocol: RNA Cleanup and Concentration (Qiagen). Finally, the RNA was eluted twice in 14 μl nuclease-free water. The yield and CyDye incorporation were measured with the NanoDrop ND-1000 (Thermo Scientific).

### Microarray hybridization, scanning & data processing

Each hybridization mixture was made up from 300 ng 'Test' and 300 ng 'Reference' sample according to the Two-Color Microarray-Based Gene Expression Analysis Manual version 5.5 (Agilent Technologies). The RNA was allowed to hybridize for 16 hours at 65°C and 10 RPM. Afterwards the slides were washed and scanned in an ozone-free room with the Agilent DNA microarray scanner G2565BA (Agilent Technologies). Slides were scanned with eXtended Dynamic Range and 5 μm resolution. Microarray data was extracted with Feature Extraction Software version 9.5.3 (Agilent Technologies). The obtained median signals were log_2 _converted and the average log_2 _signal intensity was calculated for the foreground and background signals. Log_2 _converted ratios were used to perform standard PCA and median signal intensities were used for the calculation of Spearman correlations between samples.

## Competing interests

The authors declare that they have no competing interests.

## Authors' contributions

MdJ and TMB conceived the method and wrote the paper; MdJ developed the method; MdJ and JV performed the experiments; OB and HR analyzed the data; TMB and HPS supervised the project. All authors read and approved the final manuscript.

## Supplementary Material

Additional file 1 **RNA quality Single and Semi-single samples**. This figure shows the RNA profiles of the Single and Semi-single samples as given by the Agilent 2100 BioAnalyzer together with their respective RNA Integrity Numbers (RIN). Click here for file

Additional file 2 **RNA quality Development samples**. This figure shows the RNA profiles of the Development samples as given by the Agilent 2100 BioAnalyzer together with their respective RNA Integrity Numbers (RIN). Click here for file
