# Supplementary figures and images for: RNA isolation method for single embryo transcriptome analysis in zebrafish
Source: BMC Res Notes. 2010 Mar 16;3:73. doi: 10.1186/1756-0500-3-73 (PMC2845602; doi:10.1186/1756-0500-3-73)

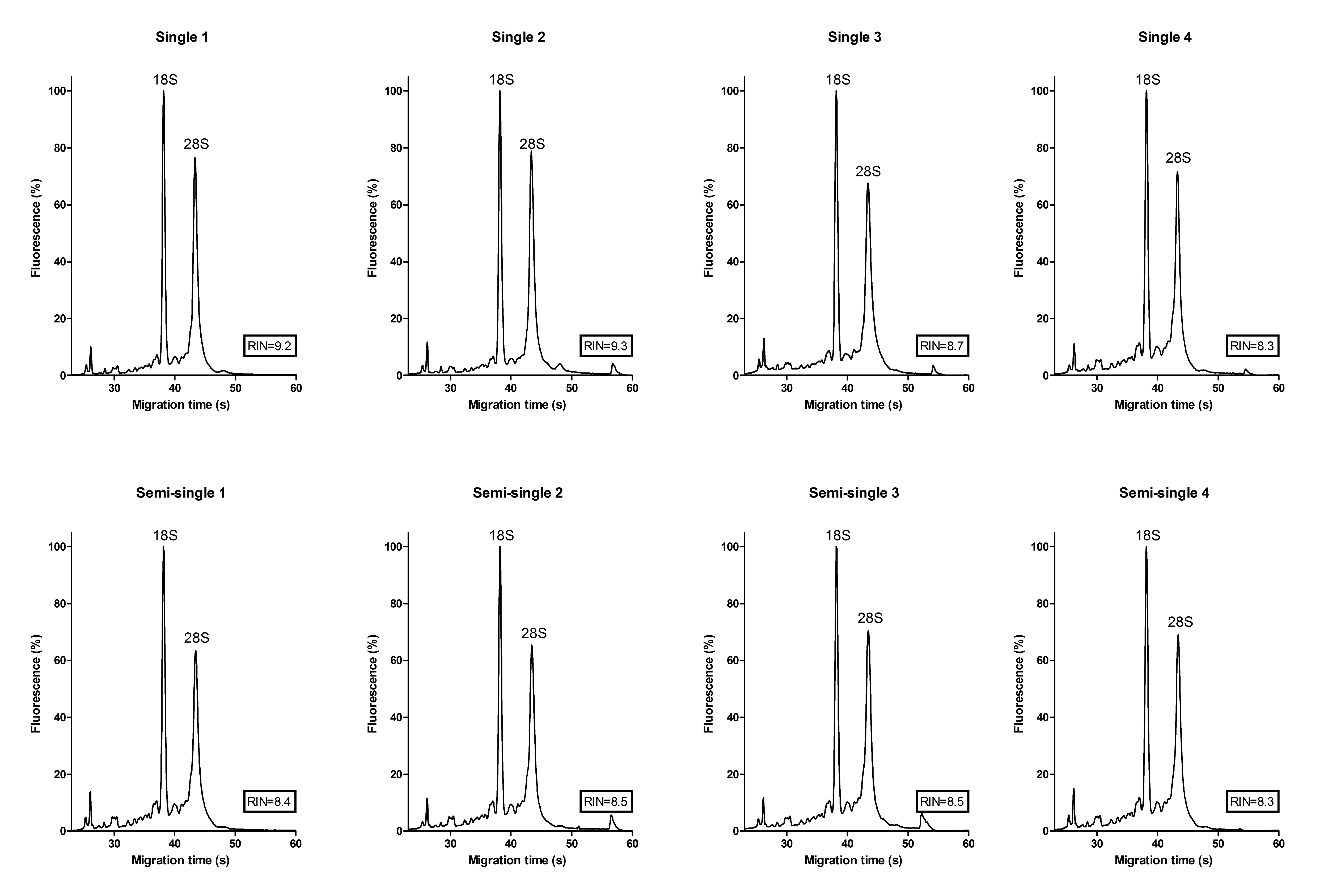

Supplement: Additional file 1 — RNA quality Single and Semi-single samples. This figure shows the RNA profiles of the Single and Semi-single samples as given by the Agilent 2100 BioAnalyzer together with their respective RNA Integrity Numbers (RIN). [file 1756-0500-3-73-S1.TIFF]

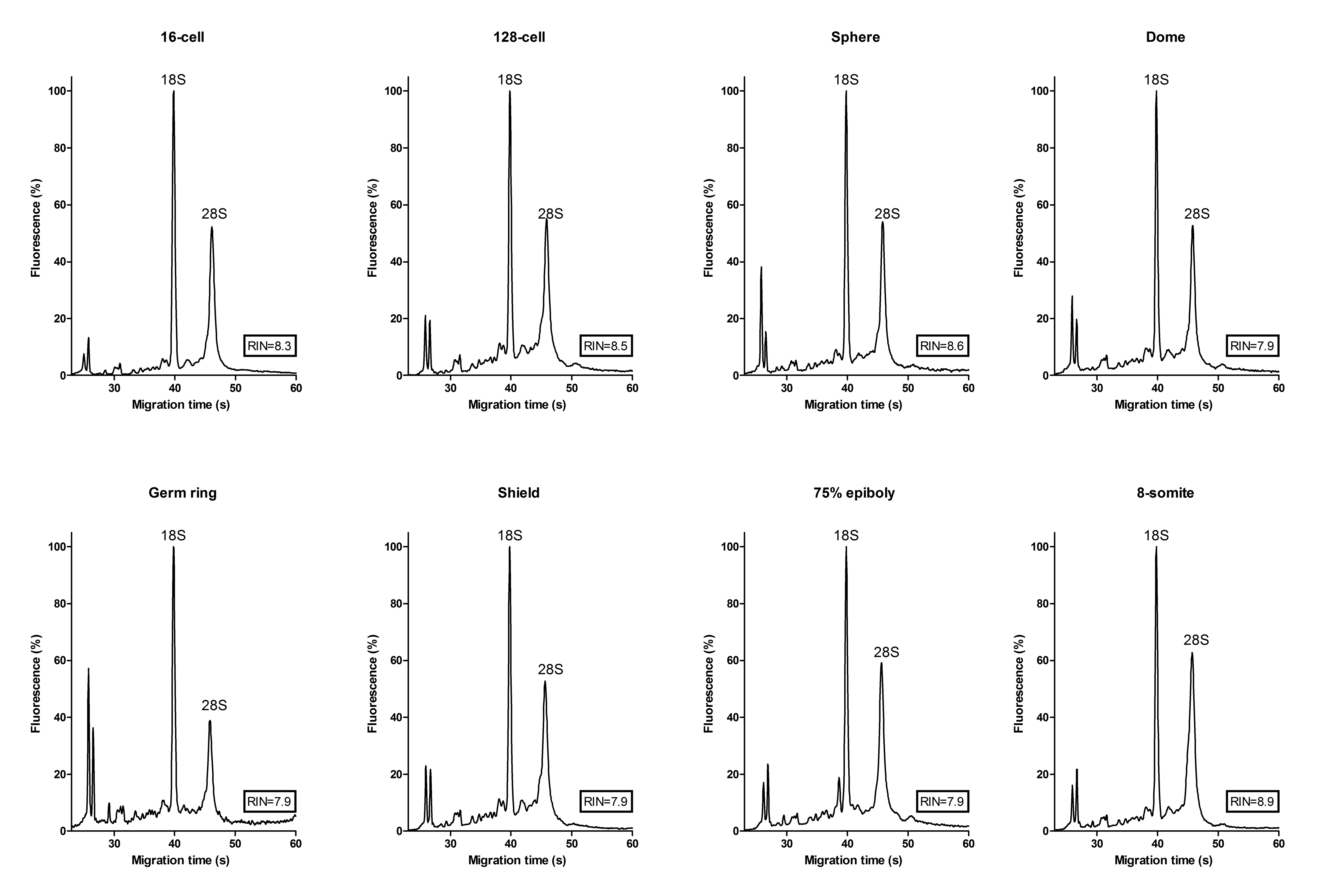

Supplement: Additional file 2 — RNA quality Development samples. This figure shows the RNA profiles of the Development samples as given by the Agilent 2100 BioAnalyzer together with their respective RNA Integrity Numbers (RIN). [file 1756-0500-3-73-S2.TIFF]
